# Comparing the Diagnostic Accuracy of Ultrasonography, CT, MRI, and PET/CT in Cervical Lymph Node Metastasis of Oral Squamous Cell Carcinoma

**DOI:** 10.3390/biomedicines11123119

**Published:** 2023-11-22

**Authors:** Masaki Takamura, Yutaka Nikkuni, Takafumi Hayashi, Kouji Katsura, Hideyoshi Nishiyama, Manabu Yamazaki, Satoshi Maruyama, Jun-ichi Tanuma

**Affiliations:** 1 Division of Oral and Maxillofacial Radiology, Faculty of Dentistry & Graduate School of Medical and Dental Sciences, Niigata University, 2-5274 Gakkocho-dori, Chuo-ku, Niigata 951-8514, Japan; 2 Division of Oral Pathology, Faculty of Dentistry & Graduate School of Medical and Dental Sciences, Niigata University, 2-5274 Gakkocho-dori, Chuo-ku, Niigata 951-8514, Japan; 3 Oral Pathology Section, Department of Surgical Pathology, Niigata University Medical & Dental Hospital, 1-754 Asahimachi-dori, Chuo-ku, Niigata 951-8520, Japan

**Keywords:** oral cancer, lymph node metastasis, multidetector computed tomography, magnetic resonance imaging, PET-CT

## Abstract

(1) Background: In oral cancer staging, ultrasonography (US), computed tomography (CT), magnetic resonance imaging (MRI), and 2-deoxy-2-[fluorine-18]fluoro-D-glucose (FDG) with positron emission tomography/computed tomography (PET/CT) are routinely used in clinical practice. The present study is a retrospective examination of the diagnostic accuracy of cervical lymph node metastasis using US, CT, MRI, and PET/CT, with histopathological diagnosis as a reference, to compare the different diagnostic imaging modalities. (2) Methods: The participants included 16 patients with oral squamous cell carcinoma who underwent US-, CT-, MRI-, and PET/CT-based preoperative diagnostic imaging and simultaneous primary lesion resection and neck dissection, including 82 level regions and 424 lymph nodes. We compared the sensitivity, specificity, accuracy, positive predictive value, and negative predictive value of each imaging modality based on the imaging results and the pathology results of metastasis. (3) Results: Of the four diagnostic imaging modalities, PET/CT exhibited the highest sensitivity but the lowest specificity and accuracy. US, CT, and MRI had high specificities. Comparing each level region and lymph node showed that differences were observed in PET/CT. (4) Conclusions: PET/CT to diagnose lymph node metastasis requires a comprehensive evaluation because it produces more false positives than other diagnostic imaging modalities. Using US, CT, and MRI, which have excellent spatial resolution, improves diagnostic accuracy at the lymph node level.

## 1. Introduction

The head and neck region holds at least one-third of all lymph nodes in the entire body [1]. It is common for oral squamous cell carcinoma (OSCC) to have cervical lymph node metastasis, with even early-stage patients exhibiting lymph node metastasis at a rate of approximately 23% [2]. Whether an OSCC patient has lymph node metastasis is important in terms of their prognosis, and there is clinical significance to an accurate preoperative diagnosis of whether there is lymph node metastasis. Reports encourage aggressive neck dissection if the risk of late-onset lymph node metastasis exceeds 20% [3]. In recent years, the incorporation of depth of invasion into the T classification of primary lesions in OSCC staging has been observed as being useful in predicting such risks. There is some controversy over the use of prophylactic neck dissection [4,5,6], with no clear conclusions reached, and use depends largely on each respective institution’s policies.

In addition to palpation, methods used to diagnose lymph node metastasis also include diagnostic imaging modalities such as CT, MRI, US, and PET/CT. Many prior reports have compared these different methods [7,8,9,10,11,12,13,14,15,16,17,18,19,20], and although the American Joint Committee on Cancer/Union for International Cancer Control (AJCC/UICC) 8th edition also mentions diagnostic imaging [21], the characteristics of each diagnostic imaging modality in preoperative evaluation remain inadequately studied, as do the approaches to compensate for their weak points. There have been some meta-analyses published regarding the diagnostic accuracy of each diagnostic imaging modality [7,8,10], but these were relatively crude studies using individual patients or dissection sides. Therefore, no adequate study has been conducted on each lymph node, which would be essential for any detailed comparison. Clinicians may be interested in the diagnostic accuracy of each region, while radiologists may focus on the diagnostic accuracy of individual lymph nodes. Comparing the diagnostic accuracy of each lymph node not only addresses clinicians’ interests but is also considered significant from a fundamental medical perspective. Furthermore, there is a paucity of studies that have simultaneously investigated these two factors [22]. Moreover, remarkable advances in diagnostic imaging equipment in recent years have enabled evaluations using images with higher spatial resolution, tissue contrast and less noise than in past reports, and it is now possible to image small lymph nodes that could not be imaged. This study aimed to retrospectively compare the diagnostic accuracy of each diagnostic imaging modality. We evaluated their diagnostic accuracy in relation to cervical lymph node metastasis in preoperative diagnostic imaging for OSCC, level by level and lymph node by lymph node, with histopathological diagnosis as the reference.

## 2. Materials and Methods

### 2.1. Patients

Sixteen patient participants who were diagnosed with OSCC in Niigata University Medical & Dental Hospital between April 2016 and March 2021 underwent all the preoperative imaging tests, US, CT, MRI, and PET/CT, and underwent primary lesion resection and neck dissection, including 82 dissected level regions and 424 dissected lymph nodes. Patients who had undergone preoperative chemotherapy or preoperative radiation therapy were excluded, as were those in whom renal dysfunction or alike prohibited the use of contrast-enhanced examination. In patients for whom multiple preoperative examinations were performed, evaluation was conducted using the initial images.

The participants comprised nine men and seven women, with a mean age of 67.6 years (age range, 23–87 years). The primary site was the tongue for nine patients, mandibular gingiva for four patients, maxillary gingiva for two patients, and buccal mucosa for one patient. Neck dissection was performed on the affected side only in 13 patients, with three cases undergoing bilateral dissection. As a result, a total of 82 regions were covered: 19 regions in level I, 18 in each of levels II to IV, and 9 in level V. For clinical N classification, nine cases were N1, five were N2b, and two were N2c. The number of days from examination to surgery was as follows: maximum 71 days, minimum 7 days, on average 31.5 days for US; maximum 64 days, minimum 9 days, on average 32.4 days for CT; maximum 49 days, minimum 8 days, on average 23.5 days for MRI; and maximum 51 days, minimum 5 days, on average 25.6 days for PET/CT.

This study was approved by the Ethics Committee of Niigata University Medical and Dental Hospital (No. 2021-0085), and informed consent was obtained as an opt-out approach on our institutional website, on which information concerning the study objectives and procedures was published, instead of using written informed consent.

### 2.2. Imaging Protocol

US was performed by a dental radiologist with at least 5 years of experience, using the ultrasound diagnosis device HI VISION Preirus (Fujifilm Healthcare, Japan, Tokyo, Japan) and an electronic linear probe (L64) at a frequency of 5 to 18 MHz to scan the left and right cervical lymph nodes. Still images of lymph nodes that were reproducible at a short diameter of 3 mm or greater were preserved in the cross-section that had the greatest size in transverse and longitudinal views. Doppler and strain elastography were also used as needed.

CT images were taken using a 64-row multi-row detector CT (Ingenuity Elite; Philips Medical Systems, Amsterdam, The Netherlands) for 12 patients and 320-row area detector CT (Aquilion ONE, CANON Medical Systems, Otawara, Japan) for 4 patients. Tube voltage was 120 kV with automatic adjustment of the tube current, with a slice thickness of 1 mm, a slice interval of 1 mm, an FOV of 220 mm, and a pitch factor of 0.391 with the Ingenuity Elite and 0.637 with the Aquilion ONE. Where metal restoration was found in the mouth, contrast-enhanced imaging was followed through additional imaging with the gantry tilted to be parallel to the inferior border of the mandible. Coronal multiplanar reconstructed images at 2 mm intervals were created from the axial section images.

MRI was performed at 1.5 T (Excelart Vantage Titan, CANON Medical Systems) in 10 cases and 3.0 T (Discovery MR750w, GE Healthcare, Chicago, IL, USA) in 6 cases. Axial and coronal fat-suppressed T2-weighted images and axial and coronal fat-suppressed contrast-enhanced T1-weighted images were used, with a slice thickness of 5 mm and slice gap of 0 mm with the 1.5 T device and a slice thickness of 5 mm and slice gap of 1 mm for coronal sections. The 3.0 T device had a slice thickness of 5 mm and a slice gap of 1 mm and a slice thickness of 4 mm and a slice gap of 1 mm for coronal sections.

PET/CT imaging was performed using Biograph mCT Flow (Siemens Healthineers, Erlangen, Germany). Patients received 199.4–290.6 MBq of FDG after at least 6 h of fasting, with blood glucose levels at or below 200 mg/dL. PET/CT imaging was initiated at approximately 60 min after the radioisotope was administered. The voxel size was 4.07 mm × 4.07 mm × 2 mm.

### 2.3. Evaluation of Lymph Nodes

All levels of cervical lymph nodes were included: I to V. The positive diagnostic criteria for metastasis were a comprehensive evaluation of the short diameter of the lymph node (US: 8 mm or more at levels I and II, or 6 mm or more in other regions; CT and MRI: 10 mm or more) [18,23], morphology (whether there is a hilum, including the long/short ratio), and the internal condition (heterogeneity associated with keratinization or internal necrosis). In addition to the above, US involved the evaluation of abnormalities or defects in the course of blood vessels via Doppler, centered on the lymphatic portal area, or blood flow signals at the margins, with evaluation of the hardness compared to the surrounding tissue in strain elastography, which were judged comprehensively along with B-mode findings [20,21]. PET/CT included visual evaluation of whether there was FDG accumulation in the lymph nodes as compared to the surrounding tissue; in cases of uncertainty, a decision was made by consulting the SUVmax, which is the maximum value of the standardized uptake value (SUV) within the region of interest (threshold value: 2.5) [13,22].

Two dental radiologists (15 years and 5 years of experience) independently evaluated for lymph node metastasis in each cervical region. The evaluation was performed with each of the diagnostic imaging modalities, with concealment of the patient’s basic information and diagnostic results, among others. Where the two had different evaluations, a decision was reached by consensus.

Lymph nodes that were resected and dissected from the neck dissection were fixed in 10% formalin solution and hematoxylin and eosin-stained using the usual method. An oral pathologist conducted a histopathological evaluation for any metastasis.

The examination of lymph node by lymph node was performed by comparing and matching size and morphology on images and in pathological specimens with reference to intraoperative findings. The diagnostic accuracy of each diagnostic imaging modality was found level by level and lymph node by lymph node.

### 2.4. Statistical Analysis

A 2 × 2 table was created from the results of diagnostic imaging and the results of the histopathological diagnosis regarding whether there was any metastasis, and the sensitivity, specificity, accuracy, positive predictive value, and negative predictive value were found for each diagnostic imaging modality. Accuracy, positive predictive value, and negative predictive value are expressed as follows:

(1) $$\mathrm{accuracy}=\frac{TP+TN}{TP+FP+FN+TN}$$

Abbreviations: TP—true-positive; FP—false-positive; FN—false-negative; TN—true-negative.

(2) $$\mathrm{positive}\;\mathrm{predictive}\;\mathrm{value}=\frac{TP}{TP+FP}$$

(3) $$\mathrm{negative}\;\mathrm{predictive}\;\mathrm{value}=\frac{TN}{FN+TN}$$

Wilson’s score method was used for 95% confidence limits. McNemar’s test was used to compare the diagnostic imaging modalities. The Kruskal–Wallis test and Mann–Whitney U test were used for statistical examination of the period from the date of each imaging examination until surgery. The level of statistically significant difference was set to *p* < 0.05. All statistical analyses were performed using Statistical Package for the Social Sciences (IBM Japan Inc., Tokyo, Japan).

## 3. Results

Of the 424 lymph nodes that were included, 22 were histopathologically positive for metastasis. In terms of the level regions, out of 82 regions, 18 had lymph nodes histopathologically positive for metastasis. This includes ten regions for level I, four regions for level II, three regions for level III, and one region for level IV. In terms of neck side, out of 19 neck sides, 11 were histopathologically positive for metastasis.

The number of lymph nodes deemed positive for metastasis was 13 with US, 14 with CT, 12 with MRI, and 33 with PET/CT. Of these, 13 out of 13 with US, 14 out of 14 with CT, 12 out of 12 with MRI, and 30 out of 33 with PET/CT could be matched to dissected lymph nodes. The three lymph nodes that could not be matched in PET/CT were all from samples that were histopathologically negative for metastasis. Therefore, these three lymph nodes were all treated as false positives. As a result, there were 11, 11, 9, and 14 true positives, respectively, and 2, 3, 3, and 19 false positives, respectively.

With lymph nodes deemed negative for metastasis on imaging despite being histopathologically positive for metastasis, it was possible to match four lymph nodes from US, five from CT, four from MRI, and one from PET/CT. In turn, there were seven, six, nine, and seven lymph nodes histopathologically positive for metastasis that could not be matched for reasons such as an extremely small size of the lymph nodes or metastatic foci or multiple candidates, and these were also treated as false negatives.

**Figure 1:**
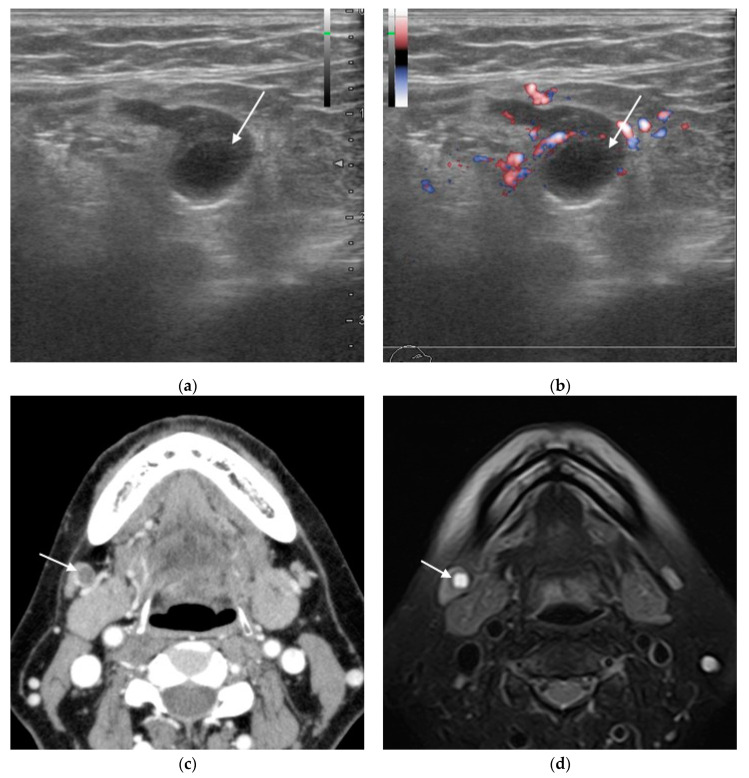

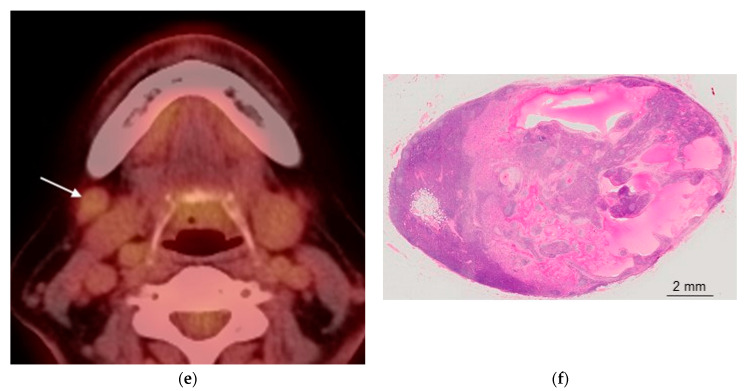
Woman in her 50s, right-side submandibular lymph node with right-side tongue squamous cell carcinoma (histopathologically positive for metastasis). (**a**) B-mode ultrasonography (US) sagittal section image; (**b**) Doppler US sagittal section image; (**c**) contrast-enhanced computed tomography (CT) transverse image; (**d**) fat-suppressed T2-weighted transverse image; (**e**) 2-deoxy-2-[fluorine-18]fluoro-D-glucose with positron emission tomography CT (18F-FDG PET/CT) transverse image; (**f**) hematoxylin and eosin (H&E)-stained histopathology image. All diagnostic imaging modalities were positive for metastasis. US shows a hypoechoic area (arrow) internally, with CT and magnetic resonance imaging (MRI) showing a focal defect (arrow). Histopathology showed loss of lymph node structure in the metastatic tumor region, with accumulation of an acidophilic mucus-like substance.

**Figure 2:**
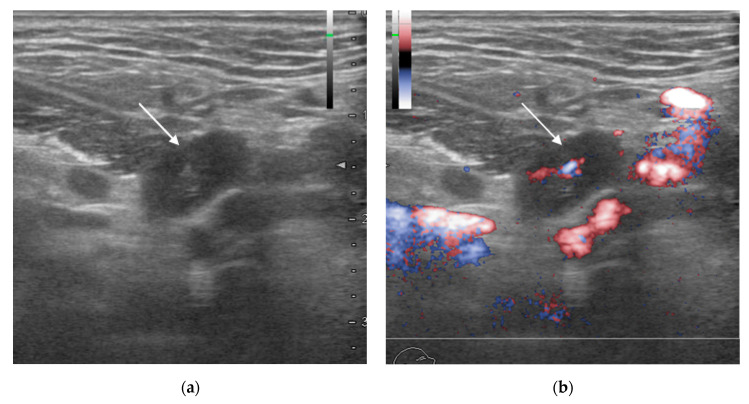

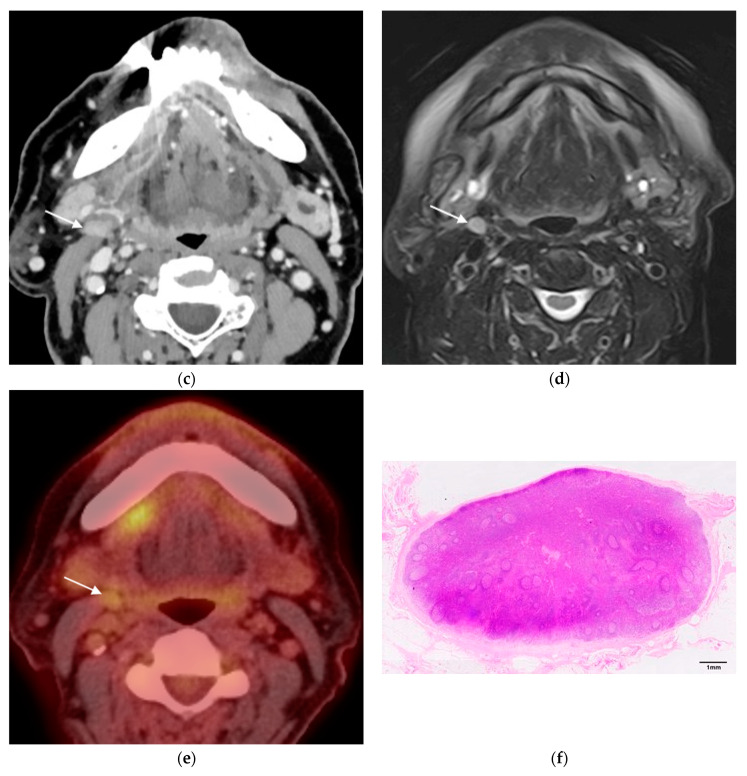
Man in his 70s, right-side superior internal jugular lymph node with squamous cell carcinoma of the right-side mandibular gingiva (histopathologically negative for metastasis). (**a**) B-mode ultrasonography (US) transverse image; (**b**) Doppler US transverse image; (**c**) contrast-enhanced computed tomography (CT) transverse image; (**d**) fat-suppressed T2-weighted transverse image; (**e**) 2-deoxy-2-[fluorine-18]fluoro-D-glucose with positron emission tomography CT (18F-FDG PET/CT) transverse image; (**f**) hematoxylin and eosin (H&E)-stained histopathology image. US, CT, and magnetic resonance imaging (MRI) were negative for metastasis (arrow); 18F-FDG PET/CT was positive for metastasis considering the degree of fluorodeoxyglucose accumulation (arrow). Histopathology showed follicular hyperplasia, but no tumor metastasis.

**Figure 3:**
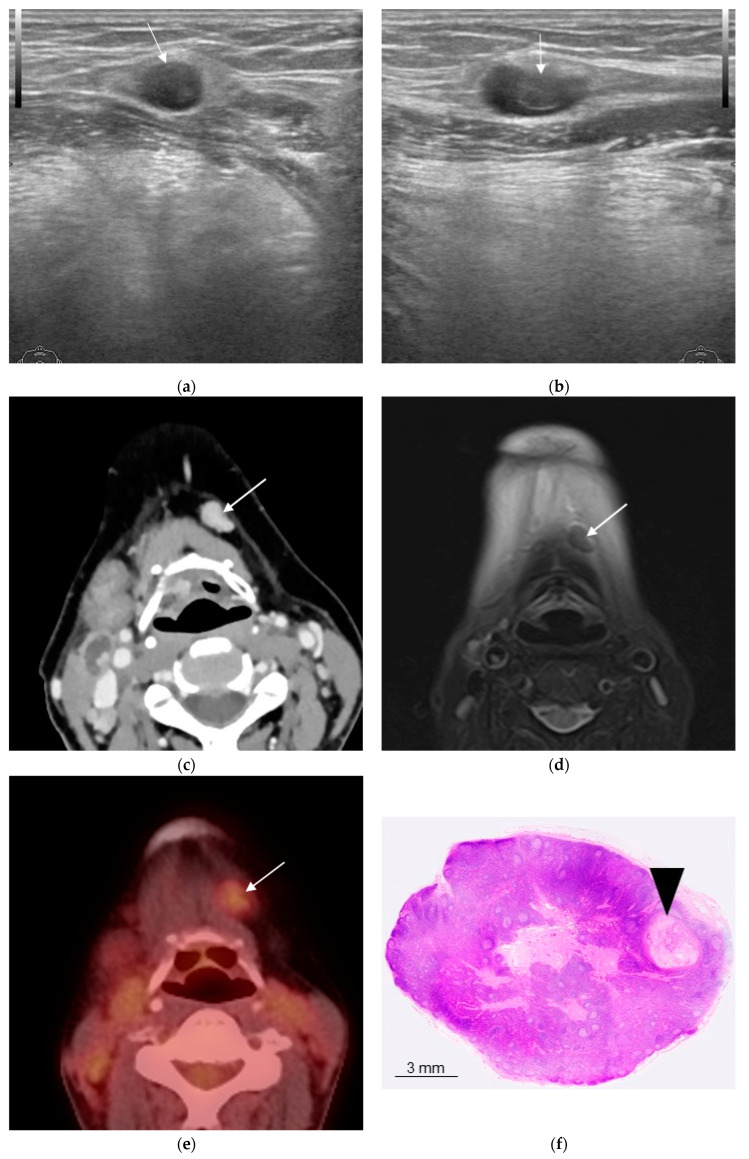
Woman in her 50s, left-side submental lymph node with right-side tongue squamous cell carcinoma (histopathologically positive for metastasis). (**a**) B-mode ultrasonography (US) coronal section image; (**b**) B-mode US sagittal section image; (**c**) contrast-enhanced computed tomography (CT) transverse image; (**d**) fat-suppressed T2-weighted transverse image; (**e**) 2-deoxy-2-[fluorine-18]fluoro-D-glucose with positron emission tomography CT (18F-FDG PET/CT) transverse image; (**f**) hematoxylin and eosin (H&E-stained histopathology image. US, CT, and magnetic resonance imaging (MRI) were negative for metastasis (arrow); 18F-FDG PET/CT was positive for metastasis considering the degree of fluorodeoxyglucose accumulation (arrow). Histopathology showed a metastasis with a diameter of 2 to 3 mm in the lymph node interior (arrow head).

Although almost none of the lymph nodes that were negative for metastasis in both imaging and pathology could be matched one-to-one with the images, all were treated as true negatives because this followed the matching of the lymph nodes that were positive for metastasis. Table 1 shows the results of the lymph node matching process. Three cases diagnosed with or without metastasis by each modality are shown (Figure 1, Figure 2 and Figure 3).

Table 2 shows the diagnostic accuracy of lymph node metastasis at the regional neck level. For each level region, US had a sensitivity, specificity, accuracy, positive predictive value, and negative predictive value of 50.0%, 96.9%, 86.6%, 81.8%, and 87.3%, respectively. With CT, these were 55.6%, 95.3%, 86.6%, 76.9%, and 88.4%, respectively. With MRI, these were 44.4%, 95.3%, 84.1%, 72.7%, and 85.9%, respectively. With PET/CT, these were 77.8%, 79.7%, 79.3%, 51.9%, and 92.7%, respectively.

Table 3 shows the diagnostic accuracy of lymph node metastasis on a lymph node basis. For each lymph node, US had a sensitivity, specificity, accuracy, positive predictive value, and negative predictive value of 50.0%, 99.5%, 96.9%, 84.6%, and 97.3%, respectively. With CT, these were 50.0%, 99.3%, 96.7%, 78.6%, and 97.3%, respectively. With MRI, these were 40.9%, 99.3%, 96.2%, 75.0%, and 96.8%, respectively. With PET/CT, these were 63.6%, 95.3%, 93.6%, 42.4%, and 98.0%, respectively.

McNemar’s test showed that PET/CT had a statistically significant difference from US, CT, and MRI (*p* < 0.05). No statistically significant differences were found among US, CT, and MRI.

The Kruskal–Wallis test showed no statistically significant differences between US, CT, MRI, and PET/CT in terms of the time from examination to surgery. The Mann–Whitney U test showed no statistically significant differences for any of the examinations (*p* < 0.05).

## 4. Discussion

The present study used histopathological diagnosis as a reference to examine the diagnostic accuracy of US, CT, MRI, and PET/CT on metastatic lymph nodes by level region and by lymph node. We found that PET/CT exhibited high sensitivity but did not always detect metastatic lymph nodes precisely.

There have been various reports on the diagnostic of lymph node metastasis, including patient-based [7,8,12], left-/right-based [7,8,17], region-level-based [7,8,9,13,16,17,22], and lymph node-based methods [12,14,15,18,19,20,22,24,25,26,27]. Lymph node-based methods of study appear to be the most accurate techniques for evaluating the diagnostic accuracy of each diagnostic imaging modality. The limitations of spatial resolution of each diagnostic imaging modality, however, make it virtually impossible to image all lymph nodes. Additionally, one-to-one histopathological matching is essentially impossible. The present study involved matching a limited number of lymph nodes that could be imaged with pathological specimens to the extent possible using size, morphology, and internal structure as references. Moreover, the large number of lymph nodes that could not be matched was classified based on interpretation, as described above. Comparison of each region level is a simple technique that is therapeutically useful from a dissection region-based perspective. However, this is not a quantified method for measuring diagnostic accuracy. These inaccuracies can result in false positives and negatives being mixed with true positives from region to region. Although perfect accuracy is not achievable, the present study was intended to be both region-level-based in terms of clinical usefulness and lymph node-based in terms of diagnostic accuracy.

PET/CT exhibited the highest sensitivity and negative predictive value of the diagnostic imaging modalities studied here but also had the lowest specificity and accuracy and the greatest number of false positives. Possible reasons for this include overly sensitive detection, including not only metastatic tumor nodes but also histopathological changes of non-metastatic tissue in lymph nodes, such as follicular hyperplasia, resulting in a greater number of false positives (Figure 2) [28]. Conversely, there was also a patient in whom only PET/CT was able to detect a lymph-node metastasis that the other diagnostic imaging modalities missed, as well as a patient in whom high diagnostic accuracy was observed (Figure 3). However, this patient had only a metastatic focus at approximately 2–3 mm, the false-positive tendency in which PET/CT over-detects accumulation of the surrounding non-metastatic tissue consequently may have provided a true positive. Hence, it was not suggested that PET/CT always exhibited higher diagnostic accuracy than other diagnostic modalities.

The FDG used for PET/CT here was to image a reflection of glucose metabolism; physiological accumulation is also known to happen in the tonsils, along with accumulation in inflammatory lesions [28]. Various factors cause lymph nodes that have experienced follicular hyperplasia to reactively enhance their glucose metabolism, increasing FDG accumulation and hindering distinguishing metastatic tumors and follicular hyperplasia. Reports evaluating lymph node metastasis via PET/CT have employed semi-quantitative values such as SUVmax [22,24,25,29]. In the present study, an SUVmax of 2.5 or higher was deemed positive for metastasis, considering that this threshold value is relatively common. Tumor cells comprise varying proportions of the interiors of lymph nodes that are positive for metastasis; thus, measured values are not necessarily reflective of the glucose metabolism of the tumor cells. Reportedly, there is also a correlation between the SUVmax of a lymph node and the number of secondary follicles. Hence, FDG accumulation has been shown in non-metastatic lymph nodes [30]. Conversely, metastatic lymph nodes with significant internal necrosis show no FDG accumulation in some instances [28]. Although PET/CT allows for an objective evaluation using a semi-quantitative value, blindly addressing only the threshold value without evaluating the overall picture naturally leads to misdiagnosis; thus, it should only be used as an aid. Several studies have proposed different threshold values for each level region and for respective short diameters of the lymph nodes [15], and it may be necessary to use different ones for different circumstances. As already noted, PET/CT exhibited the highest negative predictive value of the diagnostic imaging modalities considered here. The total absence of any FDG accumulation may largely rule out not only tumor metastasis but also reactive changes such as follicular hyperplasia. From this perspective, the absence of any FDG accumulation in regional lymph nodes may be a very powerful criterion in determining whether neck dissection is indicated [25].

PET/CT had a sensitivity of 77.8% when examined level region by level region, but a difference was found at 63.6% when it was examined lymph node by lymph node. This may be attributed to a lymph node exhibiting significant FDG accumulation observed within a region, and this accumulation may have captured a non-metastatic reactive lymph node in the same region and not necessarily a metastatic lymph node. This phenomenon is likely to be encountered by many radiologists, and it is interesting in terms of diagnostic radiology, but it seems to be overlooked because it does not affect treatment strategy in many cases. Even if multiple enlarged lymph nodes are found within a region, it is difficult to accurately diagnose on an individual basis. The criteria of assuming metastasis in a region where groups of three or more borderline nodes are found has been proposed [31]; however, this requires revision in light of advances in instrument performance compared to that in the 1990s when the report was published.

US, CT, and MRI, meanwhile, tended to have high specificity and accuracy despite having a lower sensitivity than PET/CT. On both a level region basis and a lymph node basis, accuracy exceeded 80% with US, CT, and MRI. Therefore, these methods have sufficient diagnostic accuracy for use in preoperative diagnosis. US and CT exhibited largely similar trends and exhibited high positive predictive values. US and CT have a higher spatial resolution than PET/CT, enabling more accurate detection of morphological changes and internal necrosis, which may have led to the high positive predictive values.

Considering lymph nodes compared to level region evaluations, MRI and PET/CT provided slightly inferior results, whereas US and CT tended to be largely similar on a lymph node basis. One study concluded that MRI has either equivalent or superior diagnostic accuracy compared to CT [14]. As already noted, they have a large slice thickness of 4–5 mm, and the limitation of spatial resolution may hinder the detection of small metastatic lymph nodes. In recent years, studies have reported the use of devices that improved spatial resolution in MRI and PET/CT, and further improvements to diagnostic accuracy are expected in the future [32,33].

Some studies comparing diagnostic imaging and fine-needle aspiration cytology (FNAC) have also been reported [34,35,36]. While the diagnostic accuracy is high, it is not sufficient to replace diagnostic imaging. Although FNAC can aid in lymph node diagnosis, it should be noted that its accuracy depends on the operator’s experience, and there is a tendency for false negatives due to its technical nature.

The present results showed similar trends to past reporting, but imaging conditions, including slice thickness, varied in every report. Therefore, simple comparisons based solely on numerical values should be avoided when compared against past reports.

The diagnostic imaging modalities used in the present study showed a certain number of false positives and false negatives. Especially, false negatives are expected in micrometastases that do not meet the spatial resolution of the diagnostic imaging modalities. These instances also occur when a judgment of metastasis cannot be reached, even if the tumor has replaced most of the lymph node interior. This tendency was particularly prevalent outside of PET/CT. Often, this trend is observed with poorly differentiated OSCC, which may potentially not be detected as a pathological change in a tumor node if not accompanied by marked necrosis or keratinization. Moreover, metastatic lymph nodes smaller than the spatial resolution cannot be detected to begin with; therefore, a certain number of false negatives will be inevitable regardless of the diagnostic imaging modality used. Moreover, the lymphatic network should be considered as a unit rather than as individual lymph nodes. Even at a larger level, it has been difficult to detect lymph node metastases at short diameters of less than 10 mm [22]. According to reports using immunostaining and other methods, approximately 10% of cases were found to have micrometastases that could not be detected through the use of conventional methods [37,38,39,40].

The results of this study have great implications for the daily clinical practice of oral squamous cell carcinoma. PET/CT images glucose metabolism within lymph nodes, whereas US, CT, and MRI image lymph nodes interior, and each has different characteristics. In daily clinical practice, when it is difficult to diagnose the presence or absence of lymph node metastasis, we advocate considering the findings of US, CT, and MRI, which have the highest spatial resolution, in addition to PET/CT. In many facilities, as in this study, the modality with the best spatial resolution is likely to be US or CT; however, US has the disadvantages that diagnostic accuracy depends on the examiner, and the examination is relatively time-consuming [41]. This point seems to depend on the human resources at each facility. Although not used in this study, MRI diffusion-weighted imaging and apparent diffusion coefficient may add new diagnostic criteria [42]. In recent years, research on diagnosing lymph nodes using artificial intelligence and radiomics has been reported, and this may be useful in diagnosing lymph node metastasis in the future [43,44].

The present study has several limitations. The first is the small sample size due to the precondition of being a single-center study for comparison of diagnostic imaging modalities common in the same subject. The second is the diversity of primary sites and stages in the oral cavity and the time lag from examination to neck dissection, which may have impacted the differences between the image and histopathological findings. Third, because the primary lesions were in the oral cavity and significantly more lymph nodes that were histopathologically positive for metastasis were found in level I, the diagnostic criteria such as short diameter or morphology may have influenced the present results. Fourth, the one-to-one correspondences in the comparisons with individual lymph nodes are not 100% certain, as they were compared and matched based on size and morphology. Fifth, because prevalence in the population is unknown, positive predictive value, negative predictive value, and accuracy may be incorrect. Sixth, multiple lymph nodes from the same person may not be independent samples, and lymph node-based analyses have minor statistical concerns.

## 5. Conclusions

In diagnostic imaging of cervical lymph node metastasis in OSCC, PET/CT exhibited the highest sensitivity but did not always detect metastatic lymph nodes precisely. Therefore, comprehensive decisions need to be made in combination with other modalities. Conversely, results suggested that US, CT, and MRI have higher diagnostic accuracy with individual lymph nodes in the diagnostic modalities with improved spatial resolution.

## Figures and Tables

**Table 1:** Collation results with pathological specimen for each lymph node.

	One-to-One Matched Lymph Nodes				Unmatched Lymph Nodes			
Imaging Modality	TP	FP	FN	TN	TP	FP	FN	TN
US	11	2	4	58	0	0	7	342
CT	11	3	5	72	0	0	6	327
MRI	9	3	4	46	0	0	9	353
PET/CT	14	16	1	45	0	3	7	338

Abbreviations: US–ultrasonography; CT–computed tomography; MRI–magnetic resonance imaging; PET/CT–2-deoxy-2-[fluorine-18]fluoro-D-glucose with positron emission tomography/computed tomography; TP–true-positive; FP–false positive; FN–false negative; TN–true negative.

**Table 2:** Comparison of diagnostic accuracy of lymph node metastasis at the regional neck level.

Imaging Modality	TP	FP	FN	TN	Sensitivity	Specificity	Accuracy	PPV	NPV
US	9	2	9	62	50.0 (39.4–60.6)	96.9 (90.5–99.0)	86.6 (77.6–92.3)	81.8 (72.1–88.7)	87.3 (78.4–92.9)
CT	10	3	8	61	55.6 (44.8–65.8)	95.3 (88.4–98.2)	86.6 (77.6–92.3)	76.9 (66.7–84.7)	88.4 (79.7–93.7)
MRI	8	3	10	61	44.4 (34.2–55.2)	95.3 (88.4–98.2)	84.1 (74.7–90.5)	72.7 (62.2–81.2)	85.9 (76.8–91.8)
PET/CT	14	13	4	51	77.8 (67.7–85.4)	79.7 (69.7–87.0)	79.3 (69.3–86.6)	51.9 (41.2–62.3)	92.7 (85.0–96.6)

Values in parentheses indicate 95% confidence limits. Abbreviations: US–ultrasonography; CT–computed tomography; MRI–magnetic resonance imaging; PET/CT–2-deoxy-2-[fluorine-18]fluoro-D-glucose with positron emission tomography/computed tomography; TP–true positive; FP–false positive; FN–false negative; TN–true negative; PPV–positive predictive value; NPV–negative predictive value.

**Table 3:** Comparison of diagnostic accuracy of lymph node metastasis on a lymph node basis.

Imaging Modality	TP	FP	FN	TN	Sensitivity	Specificity	Accuracy	PPV	NPV
US	11	2	11	400	50.0 (45.3–54.7)	99.5 (98.3–99.9)	96.9 (94.8–98.2)	84.6 (80.9–87.7)	97.3 (95.3–98.5)
CT	11	3	11	399	50.0 (45.3–54.7)	99.3 (97.9–99.7)	96.7 (94.5–98.0)	78.6 (74.4–82.2)	97.3 (95.3–98.5)
MRI	9	3	13	399	40.9 (36.3–45.7)	99.3 (97.9–99.7)	96.2 (94.0–97.7)	75.0 (70.7–78.9)	96.8 (94.7–98.1)
PET/CT	14	19	8	383	63.6 (59.0–68.1)	95.3 (92.8–96.9)	93.6 (90.9–95.6)	42.4 (37.8–47.2)	98.0 (96.1–98.9)

Values in parentheses indicate 95% confidence limits. Abbreviations: US–ultrasonography; CT–computed tomography; MRI–magnetic resonance imaging; PET/CT–2-deoxy-2-[fluorine-18]fluoro-D-glucose with positron emission tomography/computed tomography; TP–true positive; FP–false positive; FN–false negative; TN–true negative; PPV–positive predictive value; NPV–negative predictive value.

## Data Availability

The data presented in this study are available on request from the corresponding author. The data are not publicly available due to privacy.
